# Teamwork Management and Benefit of Telemedicine in COVID-19 Outbreak Control on an Offshore Vessel in the Gulf of Thailand, Songkhla Province, Thailand: A Case Report

**DOI:** 10.3390/ijerph20105813

**Published:** 2023-05-13

**Authors:** Rujjirat Pongpattarapokin, Sarunyou Chusri, Thammasin Ingviya, Sitthichok Chaichulee, Atichart Kwanyuang, Kanakorn Horsiritham, Suebsai Varopichetsan, Smonrapat Surasombatpattana, Chutarat Sathirapanya, Pornchai Sathirapanya, Chanon Kongkamol

**Affiliations:** 1 Department of Family and Preventive Medicine, Faculty of Medicine, Prince of Songkla University, Songkhla 90110, Thailand; rujjirutbew@gmail.com (R.P.); thammasin.i@psu.ac.th (T.I.); suebsai.varop@hotmail.com (S.V.); chutaratster@gmail.com (C.S.); 2 Department of Internal Medicine, Faculty of Medicine, Prince of Songkla University, Songkhla 90110, Thailand; sarunyouchusri@hotmail.com (S.C.); sporncha@gmail.com (P.S.); 3 Air Pollution and Health Effect Research Center, Prince of Songkla University, Songkhla 90110, Thailand; 4 Institute of Biomedical Engineering, Faculty of Medicine, Prince of Songkla University, Songkhla 90110, Thailand; sitthichok.c@psu.ac.th (S.C.); atichart.k@psu.ac.th (A.K.); 5 Division of Digital Innovation and Data Analytics (DIDA), Faculty of Medicine, Prince of Songkla University, Songkhla 90110, Thailand; kanakorn.h@psu.ac.th; 6 Department of Pathology, Faculty of Medicine, Prince of Songkla University, Songkhla 90110, Thailand; pornapat19@gmail.com

**Keywords:** COVID-19, vessel quarantine, telemedicine, isolation, management

## Abstract

In May 2021, there was a COVID-19 outbreak on board a construction support ship traveling from India to Thailand. Controlling the outbreak on this offshore vessel from 11 May to 2 June 2021 was applied. This case report describes the teamwork management of COVID-19 control on the vessel in the Gulf of Thailand. We summarized the COVID-19 outbreak control process on board, including active COVID-19-infected cases (CoIC) and close contacts (CoCC) identification, isolation, quarantine, treatment, and clinical monitoring using telemedicine to report their health measurements twice daily, including emergency conditions if they occurred. Active COVID-19 cases were identified by two rounds of reverse transcription polymerase chain reaction (RT-PCR) tests in all crew members, in which 7 of 29 (24.1%) showed positive results. Both the CoIC and CoCC were strictly and absolutely isolated and quarantined on the vessel. No serious medical conditions were reported during the monitoring. The third-round RT-PCR tests were conducted, and all tested negative one week later. Teamwork management in proactive COVID-19 case identification, isolation, comprehensive treatment, and close monitoring of health conditions using telemedicine devices is beneficial for controlling the COVID-19 outbreak on board.

## 1. Introduction

The COVID-19 pandemic is a serious global health threat that has affected millions of individuals worldwide, causing a significantly high morbidity and mortality rate, as well as major socio-economic disruptions [1,2]. 

COVID-19 is transmissible by close contact through respiratory droplets, shared surface contact during the use of common facilities, and staying or working in a crowded or enclosed environment with the infected person, such as theaters, restaurants, fitness gymnasiums, or workplaces [3]. 

Due to the working environment, people who work on board vessels cruising on the sea are highly vulnerable to contracting or spreading various airborne infectious diseases. Working in the same work departments and using shared facilities such as toilets, bathrooms, or canteens, which have very limited space and number available for access, were significant risks for COVID-19 spreading on board [4]. In May 2021, there was an outbreak of COVID-19 on board an offshore construction support vessel leaving India for Songkhla Province, Thailand. At that time, several COVID-19 variants of concern (VOCs) were reported, including Alpha, Beta, and Delta variants [5]. The rapid spreading of these COVID-19 VOCs, especially the delta variant, is a major concern for Thai public health agencies. Although many guidelines for managing COVID-19 outbreaks on board a vessel were endorsed by the Ministry of Public Health of Thailand (MOPH-T) [6], the Centers for Disease Control and Prevention (CDC) [7], the World Health Organization (WHO) [8], the International Chamber of Shipping (ICS) [9], and the International Maritime Organization (IMO) [10], we had no experience managing this kind of outbreak. Herein, we describe our first operation to control the COVID-19 outbreak on board a vessel in the Gulf of Thailand. Our newly developed practical guideline to use telemedicine as a method to monitor and respond to the crew members’ clinical conditions while they were isolated on the vessel offshore was described.

## 2. Materials and Methods

### 2.1. Study Setting 

We describe the management of the COVID-19 outbreak on an offshore support vessel from 11 May to 2 June 2021. The vessel was an offshore construction vessel (OCV) equipped with a 250-ton active heave compensation (AHC) plus a 30-ton AHC auxiliary crane and sports two 2000-meter-rated Oceaneering Millennium Plus remotely operated underwater vehicles (ROV). The vessel worked under a contract with a company working in the Gulf of Thailand. There were a total of 29 crew members working on board at the time of its arrival in the Gulf of Thailand. The crew comprised nine Ukrainians, nine Poles, five Filipinos, two Croatians, one Norwegian, one Indian, one Russian, and one Spaniard, but all could communicate in English. The house on the vessel deck had five levels: three for accommodation and two for working. The accommodation capacity on the vessel was 126 beds in 54 single cabins and 36 double cabins. Each floor with cabins had shared toilets and bathrooms. At the time, the vessel had no passengers other than the crew.

### 2.2. COVID-19 Spreading Control on Board

#### 2.2.1. Case Identification at the Arrival

When the vessel arrived in Songkhla on 11 May 2021, none of the crew was allowed to disembark at Songkhla port. Both nasopharyngeal and oropharyngeal swabs for reverse transcription polymerase chain reaction (RT-PCR) were immediately conducted on all crew members by the medical team of the hospital contracted to the company according to “Disease Prevention Measures to Prevent the Spread of the COVID-19 Disease for Travelers Entering into the Kingdom of Thailand” [6]. Those with positive RT-PCR tests were diagnosed as COVID-19-infected cases (CoIC), and those with negative RT-PCR tests were classified as COVID-19 close contacts (CoCC).

#### 2.2.2. Isolation for CoIC and Quarantine for CoCC Measures on Board

The CoIC were managed to stay separately on different floors from the CoCC. The CoIC were isolated individually in each cabin. They obtained the “leaves of absence” allowance but could not come out of their rooms before permission was given from the captain. Common-use areas were absolutely closed. The CoCC assigned to support the isolation measures were obligated to wear suitable personal protective equipment (PPE), including a face mask, when carrying foods and essential daily-used items to the dropping points in the levels of the vessel house where the CoIC picked up the items at different times. 

The CoCC, according to the WHO definition and the International Chamber of Shipping (ICS) guidance [8,9], underwent a quarantine process. The CoCC were given their daily tasks scheduled by the captain while strictly maintaining physical distance (at least one meter) when working with other seafarers to the extent possible, avoiding non-essential contact with any persons, disinfecting their work areas and equipment after use, returning to their cabins immediately after working hours, remaining in their cabins during rest hours, and eating all meals in their cabins. All the crews were required to wear medical masks, wash their hands frequently, especially after completing each activity, and maintain good respiratory hygiene according to the ICS guidance in IMO circular letters [9,10]. Moreover, they could not leave their rooms without permission from the captain. CoIC and CoCC were emphasized to comply with recommended practice guidelines for preventing COVID-19 spreading, such as physical distancing, wearing face masks, and frequent hand sanitization with alcohol-based sanitizers. The quarantine process applied to CoCC aimed to prevent viral dissemination from asymptomatic CoCC [11].

The medical team of the contracted hospital and the Communicable Disease Control Office enacted the vessel quarantine and the specimen collection from the CoCC for the repeated RT-PCR tests on day 7 and day 14 of the quarantine based on the guidelines for disease control in quarantine facilities of MOPH-T [12].

#### 2.2.3. Telemedicine Usage for Health Monitoring

The Division of Digital Innovation and Data Analytics (DIDA) and the medical team of the Faculty of Medicine, Prince of Songkla University, developed the online health monitoring system. The system comprised telemedicine technology through which the medical team was able to monitor the crew’s health status onshore and collect their health reports regarding the progression and severity of the current symptoms, vital signs, and results of sit-to-stand (STS) oxygen desaturation tests conducted with both CoIC and CoCC. The instruments for measuring health indices, including a blood pressure monitor, thermometer, and pulse oximeter, were immediately delivered to the crew for their self-measuring and reporting of health statuses. 

**Figure 1:**
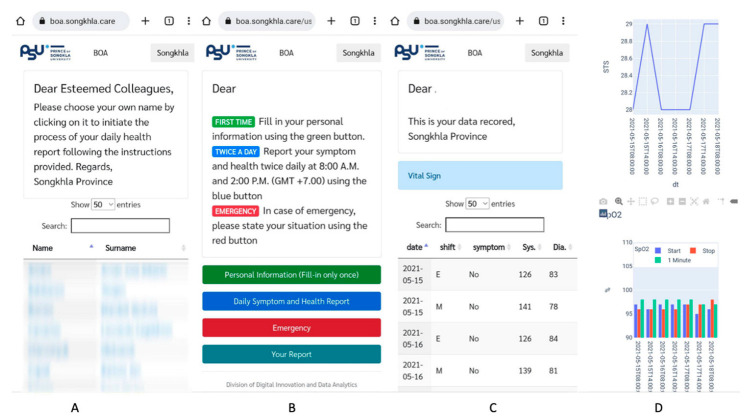
Illustrations of the online application (https://boa.songkhla.care accessed on 2 June 2021). (**A**) The page for the medical team. (**B**) The page after a successful login by a crew member. (**C**) The table shows an individual’s health results. (**D**) The graphs show an individual’s peripheral capillary oxygen saturation (SpO_2_) result in sit-to-stand test.

Health monitoring was advised to be performed twice daily throughout the period of isolation or quarantine, according to the WHO recommendation [3]. The crew members provided the data through the web applications with login security. The online application https://boa.songkhla.care (accessed on 2 June 2021) was active from 15 May to 2 June 2021. Visitors were required to enter the username ‘reviewer’ and password ‘a9f5770e33d0fab63686a9e39d20e4b1’ showed four buttons (Figure 1B). The first button allowed the provision of personal information, consisting of name, date of birth, sex, passport number, body weight, height, history of food and drug allergies, and prior or current medications. The crew members provided this information only once. The second button allowed twice-daily reporting of the symptoms, followed by a health indices report. The report included general and respiratory symptoms, systolic blood pressure, diastolic blood pressure, heart rate, body temperature, and the result of the STS test. The test was divided into three steps: before the test (record the resting oxygen saturation and heart rate), immediately after the test (record the number of rounds of STS, oxygen saturation, and heart rate), and 1 min after the test (record oxygen saturation and heart rate). STS was the test for screening pneumonia, which might complicate COVID-19. The STS test 1 min after exercise was used to evaluate exercise-induced hypoxemia, physical capacity, and exertional desaturation [13,14]. The third button was an emergency button used to notify of emergency health situations (Figure 1). In the case of abnormal health indices or emergency conditions reported, the system would automatically notify the medical team onshore through the LINE application immediately. The medical teams and the contract company would discuss the corresponding care plans. Finally, the last button was “Your report” presenting the summarized results that were reported to the medical team (Figure 1C,D).

The crew members were recommended to fill their self-reported health data to the application twice daily, i.e., at 8:00 a.m. and 2:00 p.m. They received instructions on how to use the web application to report their health status. Thence, the medical team summarized the health data on Google Sheets and coded individual person’s health status as green, yellow, or red. Green represented a crew member with a normal or abnormal health status that was clearly caused by measurement error. Yellow denoted a crew member with abnormal health reports, such as STS-induced desaturation or hypoxemia (O_2_ saturation < 96%), without clinical deterioration or health data that appeared to be measurement error. Red signified a crew member with abnormal health reports and clinical deterioration that required an immediate medical response. The Jupyter program was used to create a code for the non-reporting crew members and to generate a message to the captain asking them to report their clinical statuses and test results, and to confirm the initial results or re-test results. The medical team generated a daily summary health report with suggestions and sent it to the crew member via an intermediary. The medical team’s recommendations were based on symptoms with consideration of the individual’s STS test result and COVID-19 test status.

#### 2.2.4. Plan for Evacuation

In a medical emergency situation, the Thai Maritime Enforcement Command Center (the Royal Thai Navy) was ready to transfer the patient to the onshore hospital immediately.

#### 2.2.5. The Joint Working Group

The Songkhla Provincial Health Office (SPHO) was notified about the situation of a possible outbreak on board the vessel and asked for COVID-19 outbreak control actions on the vessel. Thereafter, a joint meeting was convened between the relevant government agencies, i.e., the Office of Disease Prevention and Control Region 12, the Faculty of Medicine, Prince of Songkla University, Songklanagarind Hospital, the contract hospital, and the employer company. The measures and practical actions to handle this outbreak were discussed and implemented later. The implemented actions followed the “Guidance for Ship Operators for the Protection of the Health of Seafarers version 3.0, issued on 20 September 2020” by the International Chamber of Shipping (ICS) in response to the coronavirus outbreak and issued initially as Circular Letter No.4204/Add.4 [10].

### 2.3. Data Collection and Analysis

We collected data on the crew members’ general characteristics, the results of health monitoring online from the Division of Digital Innovation and Data Analytics (DIDA) of the Faculty of Medicine, Prince of Songkla University the medical records of the health team that managed this outbreak, and the data from the Communicable Disease Control Office of Songkhla Port. The analysis was carried out using descriptive statistics.

### 2.4. Ethics Considerations

The ethics approval for this study was granted by the Human Research Ethics Committee, Faculty of Medicine, Prince of Songkla University (REC.65-247-9-4; date 28 June 2022). Study participants’ consent was obtained. We followed the 1964 Declaration of Helsinki and its related guidelines to conduct this study. All the study participants’ data were completely anonymous. The data were analyzed in aggregate to protect the study participants’ confidentiality.

## 3. Results

### 3.1. General Characteristics

The 29 crew members on this vessel were distributed to work in four departments: deck, engine, electro-technical, catering, and others. General characteristics, body mass index (BMI), underlying diseases, COVID-19 vaccination, their working departments, and the results of RT-PCR tests on arrival, 7 days, and 14 days afterward are shown in Table 1. A total of 7 of the 29 crew members (24.1%) tested positive for COVID-19 during this outbreak. Only one crew member had previously received the COVID-19 vaccination.

### 3.2. COVID-19 Symptoms and the Results of the RT-PCR Tests for COVID-19 upon Arrival 

None of the crew members had symptoms suggestive of COVID-19 during arrival. The day after their arrival and screening RT-PCR tests were carried out, six tested positive for COVID-19 (20.7%). The RT-PCR-positive cases were two Ukrainians, two Polish, one Indian, and one Russian. All six COVID-19 confirmed cases worked in the deck department, i.e., one second officer/DPO, two able seamen, two seaman/crane operators, and one bosun.

### 3.3. Isolation and Quarantine Process

The vessel was quarantined and anchored near Ko Nu Island, which the Marine Department of Thailand determined to be a quarantine zone. None of the crew was allowed to disembark at the Songkhla port.

The CoIC and CoCC were isolated and quarantined as previously described. 

The medical team and the captain of the vessel discussed and agreed to start Favipiravir for a 10-day course in CoIC (the only available antiviral drug in Thailand at that time). The drugs for treating COVID-19 and other medical illnesses, as well as necessary medical equipment, were delivered to the vessel on 13 May 2021 (1 day later, after the first round of RT-PCR tests were performed). The captain reported that all CoIC took the Favipiravir as prescribed.

### 3.4. Repeated RT-PCR Tests Performed

We performed the second RT-PCR tests 7 days after the first ones. One additional crew member (4.3%) had a positive RT-PCR test. There was no close contact case found from the contact case tracing performed for the last infected case.

The third RT-PCR tests were performed in the remaining CoCC 14 days after the first tests. No new case of positive RT-PCR was found in this round of testing.

### 3.5. SARS-CoV-2 Variants of Concern

After VOC analyses, there were three infected cases (42.9%) with B.1.351 (South Africa), and four infected cases (57.1%) with B.1.1.7 (United Kingdom).

### 3.6. Monitoring Health Status

**Figure 2:**
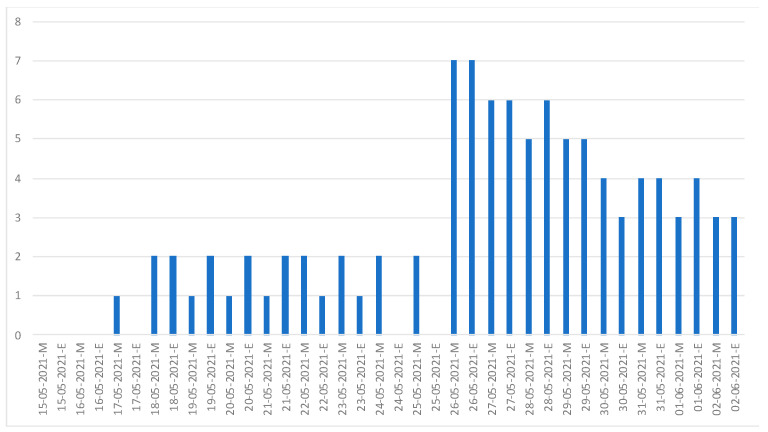
Illustrations of the number of non-responders to self-reporting from 15 May to 2 June 2021. (M: 08:00 a.m. and E: 02:00 p.m.).

Crew members cooperated well with providing self-reported health statuses. In addition, we established a group in the Line application for communication among the health team staff. After the online health monitoring systems were completed on 14 May 2021, self-reported health statuses began the next day and continued until the last confirmed case was completely isolated (2 June 2021). Personal information was provided by all crew members. During 18 days of observation with twice-daily reporting, at each report, a mean of 90.5% (26.25 of 29 crew members) self-reported their symptoms and health status (Figure 2). 

The non-responders were predominately second engineers. However, no emergency conditions appeared among them. A few days before the end of the quarantine process, the non-response rate increased. Those who were coded yellow sent an average of 5.9 reports per day due to abnormal STS indicated by desaturation or hypoxemia; however, the reported desaturations were not confirmed by repeat STS. The medical team closely monitored these individuals and observed no subsequent clinical deterioration.

### 3.7. The Summary of the Journey

The date and activities are summarized in Table 2.

## 4. Discussion

### 4.1. Principal Results

Of the 29 crew members, 7 deck crew members had asymptomatic COVID-19 infection and showed no clinical deterioration during the isolation. Six confirmed cases were found in the first RT-PCR tests, and one additional case was found in the second test. Before the quarantine ended, the third RT-PCR tests for COVID-19 were negative. Then, no new cases were identified. The crew participated well in the quarantine or isolation process. Their compliance with self-reported health statuses and favipiravir therapy in CoIC was satisfactory. Although the rate of SARS-CoV-2 virus-infected cases identified before isolation quarantine (20.7%, 6/29) on the vessel was higher than that reported in the outbreak on the Diamond Princess cruise ship (0.3%; 10/3713) [15,16], the rate of positive tests in the second PT-PCR tests under our COVID-19 control management was 4.35% (1/23), while that of the Diamond Princess cruise ship outbreak was 4.6% (172/3713) [16]. As the number of people on board during the outbreak reported here was significantly smaller than that on board the Diamond Princess, quarantine and isolation of CoIC and CoCC cases were better carried out (1 person/1 cabin during vessel isolation or quarantine). Moreover, serial RT-PCR tests for COVID-19 were performed repeatedly according to the standard guidelines, which were different from those performed during the Diamond Princess cruise ship outbreak. Significantly, early identification of CoIC decreased the possibility of disease transmission. We believed that the smaller number of people on board the vessel and the large amount of vacant accommodation space facilitated compliance with quarantine and social distancing procedures. Moreover, on board a tourist vessel such as the Diamond Princess, strict regulations such as those used in this study are difficult to apply. 

The joint operation team tried to learn from past outbreaks, including the largest outbreak of COVID-19 on board the Diamond Princess and the outbreak on the MS Artania. The former was the earliest and most significant outbreak of COVID-19 on a cruise ship, which made it challenging to control the viral transmission and prevent serious complications, including death [4,16]. One month later, a COVID-19 outbreak occurred on the MS Artania, which was successfully controlled with no significant complications reported. Ref. [17] Concurrent with the COVID-19 outbreak on the MS Artania, WHO launched interim guidance, namely “Operational considerations for managing COVID-19 cases or outbreaks on board ships” [8].

### 4.2. The Management of the Onboard Outbreak

This event was a learning experience about the rapid response of the associated agencies in Songkhla province, Thailand, such as the Royal Thai Navy, SPHO, the Office of Disease Prevention and Control Region 12, the Office of Songkhla Province, the university hospital, a contract hospital, and private companies. Preventing COVID-19-associated morbidity or mortality and spreading COVID-19 onshore were the primary aims of the current cooperation.

At the time the vessel departed from India on 1 May 2021, India had been reported as the country with the highest average daily new COVID-19 cases in the world (more than 400,000 cases/day) [18]. Furthermore, some crew members were defined as having close contact with the infected persons. Therefore, the situation of this vessel at that time was consistent with the CDC’s definition of a COVID-19 outbreak [19].

Numerous virus variant strains existed, one of which was B.1.617.2, recorded for the first time in India in October 2020. The CDC website classified B.1.617.2 as a “variant of concern (VOCs)” with increased transmissibility, potentially reduced neutralization by some emergency use authorization for monoclonal antibody treatments, and post-vaccination sera. Nevertheless, the genomic results of SARS-CoV-2 showed that 42.9% of the positive-test cases were the B.1.351/Beta variant, which is also a VOC with evidence of increased transmissibility, significantly reduced neutralization by antibodies generated during previous infection or vaccination, and reduced effectiveness of treatments and vaccines [20], including an increased risk of hospitalization and ICU admission and a higher mortality rate compared with the wild-type virus [21]. The risk of reinfection may increase with exposure to the SARS-CoV-2, B.1.351 variant strain, which was first reported in South Africa [20]. Thus, vessel quarantine was mandatory to prevent the transmission of these VOCs to the public onshore. The confirmed cases were absolutely isolated from the CoCC on this ship to distinguish the CoIC from those who were not infected [8]. All crew members were required to stay individually in the cabins immediately after the captain received an infection control report. A personal washroom for each individual was not possible. The captain scheduled the timetable for each crew member to use the bathroom, and a crew member needed to ask for permission to use the common toilet to limit the number of people allowed at the same time in the shared areas. For the cleaning and disinfection procedures for the toilet, bathing, and common areas, the crew members were suggested to clean their hands with an alcohol-based hand rub or soap for at least 20 s before and after using the room. After a single use, environmental surfaces should be cleaned thoroughly with detergent and common disinfectants after wearing gloves and a mask [9].

The management of the isolation and quarantine processes on this vessel was effective due to the crew’s compliance with every directive, especially the captain, who put his full effort into these processes. They understood and accepted that the primary purpose of vessel quarantine was to protect the public’s health onshore from contracting the virus VOCs and to monitor their health conditions closely with a rapid medical response when indicated.

We followed the “Identification of contact issue in ICS Guidance for Ship Operators for the Protection of the Health of Seafarers version 3.0” in Circular Letter No.4204/Add.4/Rev.2 of the International Maritime Organization (IMO), which stated that “If the widespread transmission was identified, then all persons on board should be considered as close contacts having had high-risk exposure” [9,10]. For the CoCC persons, the CDC and WHO recommended a 14-day quarantine protocol after exposure [22]. The recommendations of the CDC for unvaccinated individuals who were in close contact with persons with COVID-19 addressed that they should be tested for COVID-19 immediately after being identified; if a negative test result was obtained, repeated tests for COVID-19 should be considered 5–7 days after the last exposure or if symptoms developed during the quarantine because the window period before the viral nucleic acids are detectable is up to 5 days after exposure [23]. The combined nasopharyngeal and oropharyngeal swabs for RT-PCR tests in this study were shown to increase the sensitivity of detecting the SARS-CoV-2 virus, improving the reliability of the results [24].

The management of the COVID-19 outbreak on board ships is required whenever any persons on board display symptoms suggestive of COVID-19, meet the definition of a suspected case of COVID-19, or are identified as close contacts by contact case tracing. The actions include limiting exposure to other persons on board, isolating suspected cases of COVID-19, caring for suspected cases of COVID-19, cleaning and disinfecting the vessel, and the disembarkation process of suspected cases of COVID-19 [9].

Based on the CDC recommendation, persons who had positive tests for COVID-19 should be isolated for at least ten days from the day of the positive test reported [25]. In addition, the guideline on COVID-19 infection control issued by the Department of Medical Service Thailand, updated on 6 May 2021, recommended at least 14 days of isolation from the day of the positive test reported as well [12]. Thus, we decided to apply a 14-day isolation protocol to ensure that the virus had not recovered replicating competence and that there would be minimal viral shedding.

In the situation of the COVID-19 outbreak on board detailed here, the antiviral drug was prescribed to all COVID-19 confirmed cases to achieve faster viral clearance and likely lessen the second infection rate and the risk of severe complications occurrence because we were concerned that emergency evacuation of the severe cases was a difficult and high-risk operation. However, an emergency evacuation plan was readily prepared to conform to international standards.

### 4.3. The Benefit of Telemedicine

In the middle of May 2021, there was quite limited experience in COVID-19 outbreak control on-board. Offshore outbreak control is very challenging because there are difficulties in patient evaluation and monitoring. Additionally, unpredictable weather conditions during an emergency evacuation from offshore pose immense barriers and a risk for unwanted outcomes.

Telemedicine technology was deployed to facilitate the real-time evaluation of the crew’s health conditions. The system helped the medical team onshore know the updated conditions of CoIC and CoCC and follow up. For example, from the online self-report questionnaires, the medical team recognized a susceptibility case of severe COVID-19 due to his previous medical history of asthma. This case was a top priority of concern for health monitoring.

### 4.4. The Limitations and Learning Experiences of Medical Follow-Up via Telemedicine

At the beginning of the monitoring program, the medical team found that the same crew members did not report on time every day. Thus, their report timestamps were reviewed, and it was found that they reported every day, but it was different from the time scheduled. Because of the overlap between their working time and self-reported time, they were provided a suitable self-reported time, for example, 2 p.m. and 8 p.m., as shown in Figure 2, or 2 a.m. and 8 p.m. Near the end of the quarantine period, the number of non-responders in daily health reports increased. The highest non-response rate was 27.6% (8/29 crew members). This may be related to the work in preparation for the departure schedule (30 May 2021), which was the last quarantine day. 

We found a few false-positive oxygen desaturation tests. The STS test instructions were explained using messages and video demonstrations as well. Some STS results showed oxygen desaturation without any symptoms or clinical deterioration. After the discussion among the medical team staff, we concluded that such results were suspected to be measurement or STS performing errors. The repeat STS tests did not confirm oxygen desaturation.

Telemedicine is a useful tool to be adopted in the COVID-19 isolation or quarantine process. This tool relieved the burden on physicians during the COVID-19 pandemic and also protected healthcare personnel from exposure to COVID-19 during work [26]. We suggest that using this way of communication between the medical team staff and the crew is effective in clinical monitoring and evaluation during the control of COVID-19 spreading on board.

Additionally, this event pushed DIDA to establish a telemedicine system for quick response to the outbreak on a vessel. DIDA gained experience from this event and developed a new web application, namely “Songkhla Care”, which was used by 150,000 users during 2021–2022 for monitoring or reporting symptoms of COVID-19 to the healthcare team.

### 4.5. Limitations of this Study

The limitation of this study is the small number of study participants and the single experience with this issue described. However, it can be an example for managing the next outbreak on board in the future. Good cooperation among the related agencies, either health or non-health offices, is the strength and learning experience from this study in handling this kind of event.

## 5. Conclusions

The COVID-19 outbreak on board an OCV may be easier to control than that on tourist vessels because this kind of vessel has fewer people involved, no customer service aim, and the guidelines for COVID-19 control on board have already been endorsed. The past and current experiences in the tourist and working vessels, as in the current study, guide us to suggest that effective screening measures should be strongly applied, tough and competent teamwork of all agencies, the vessel crew’s compliance, and an effective telemedicine system should be a ready and timely response to any on board COVID-19 outbreak.

## Figures and Tables

**Table 1:** Characteristics of the crew, their working departments, underlying diseases, vaccination history, results of RT-PCR tests.

Characteristic	Total Crew	Deck Crew	Engine Crew	Catering and Other Departments Crew
Number	29	14	8	7
Gender, *n* (%)				
Male	27 (93.1)	14 (100)	7 (87.5)	6 (85.7)
Age years, median (IQR)	43.1 (36.9,49.2)	45.2 (37.3, 53.1)	40.6 (34.4, 44.6)	40.8 (39.8, 42.7)
Body mass index (kg/m^2^), *n* (%)				
18.5–24.9	10 (34.5)	6 (42.9)	2 (25)	2 (28.6)
25–29.9	13 (44.8)	6 (42.9)	3 (37.5)	4 (57.1)
30–34.9	6 (20.7)	2 (14.3)	3 (37.5)	1 (14.3)
Nationality, *n* (%)				
Ukrainian	9 (31)	5 (35.7)	4 (50)	0
Polish	9 (31)	6 (42.9)	2 (25)	1 (14.3)
Filipino	5 (17.2)	0	0	5 (71.4)
Croatian	2 (6.9)	0	1 (12.5)	1 (14.3)
Norwegian	1 (3.4)	1 (7.1)	0	0
Indian	1 (3.4)	1 (7.1)	0	0
Russian	1 (3.4)	1 (7.1)	0	0
Spanish	1 (3.4)	0	1 (12.5)	0
Underlying disease, *n* (%)				
Asthma	0	1 (7.1)	0	0
Vaccination, *n* (%)				
Received	0	1 (7.1)	0	0
RT-PCR for coronavirus, *n*(%)				
Detectable				
on arrival (*n* = 29)	6 (20.7)	6 (42.9)	0 (0.0)	0 (0.0)
Day 7 after quarantine (*n* = 23)	1 (4.3)	1 (7.1)	0 (0.0)	0 (0.0)
Day 14 after quarantine (*n* = 22)	0 (0.0)	0 (0.0)	0 (0.0)	0 (0.0)

**Table 2:** The important dates and activities for this event.

Date	Activities	Note
1 May 2021	The vessel departed from India.	-
11 May 2021	The vessel arrived at the Port of Songkhla in Singha Nakhon district.	
	First RT-PCR tests performed (all crew, *n* = 29)	Detectable 6/29 (20.7%)
12 May 2021	The Songkhla Provincial Health Office called for a joint meeting.	A joint working team was formed, and an action plan for COVID-19 control was implemented.
13 May 2021	Medicines and equipment were delivered to the vessel.	-
14 May 2021	Web application was started.	-
19 May 2021	Second RT-PCR tests performed (on CoCC, *n* = 23).	Detectable 1/23 (4.3%). No new CoCC found.
20 May 2021	Medicines were delivered.	-
26 May 2021	Third RT-PCR tests performed.	No new detectable result.
2 June 2021	The isolation of the last CoIC was completed.	-
9 June 2021	The COVID-19 control process ended.	-

## Data Availability

All the data and analysis methods were described in this article.
